# Requirement for prevention of periodontitis in patients with metabolic syndrome

**DOI:** 10.1186/1878-5085-5-S1-A113

**Published:** 2014-02-11

**Authors:** Anna Barmasheva, Liudmila Orekhova, Ramilya Musaeva

**Affiliations:** 1 Periodontal City Center “PAKS”, 27 Dobrolubova Prospect, Saint-Petersburg, 197198, Russia; 2 Therapeutic Dentistry Department I.P. Pavlov Saint-Petersburg State Medical University, 6/8 Lev Tolstoy Street, Saint-Petersburg, 197022, Russia

## 

There is growing evidence that suggests a relationship among periodontitis and the risk factors for cardiovascular disease, including obesity, dyslipidemia, diabetes and hypertension; their association is known as metabolic syndrome (MetS). Periodontal disease was proposed as a part of MetS (1). It was found that women with more components of MetS had a significantly higher odds ratio for greater probing depth and clinical attachment loss (2).

## Objective

of the study was to compare periodontal condition and gingiva microcirculation in patients with different MetS components and healthy volunteers without periodontal lesions.

## Study design

40 patients with MetS (aged 34-64, 38% males) were equally divided into two groups depending on the number of MetS components. In the first group were 20 patients with central obesity and 2>MetS factors (hypertension/ diabetes/ dyslipidemia/ microalbuminuria); in the second group were 20 obese patients with one MetS factor. The control group included 15 healthy volunteers (age 19-23 years; 50% males) without periodontal lesions.

## Methods

Periodontal condition of all patients was accessed using Simplified Oral Hygiene Index (OHI-S), Silness & Loe plaque index (PI), sulcus bleeding index (SBI), papillary-marginal-alveolar index (PMA), Russell periodontal index (RPI); panoramic X-ray was taken. Periodontal blood flow was measured by ultrasonic Doppler device “Minimax-Doppler-K” (“Minimax”, Saint-Petersburg) with making indirect cold test. Statistical analysis was performed using SPSS package.

## Results

Patients with MetS had poor oral hygiene (OHI-S_1_>2,3; OHI-S_2_>1,9). In 1^st^ group severe periodontitis was diagnosed in 56% of patients while in 2^nd^ group it was found only in 25% of patients. There was no significant difference in SBI and PMA between patients with MetS, however the difference was significant between patients with MetS and control group (p_1,3; 2,3_<0,001). RPI was also higher in 1^st^ group than in 2^nd^ group (p_1, 2_<0,05). Periodontal pockets were deeper in 1^st^ group than in 2^nd^ group (7,3±1,9 mm vs. 5,6±0,9 mm, p_1,2_<0,05). Patients with 2>MetS factors had a linear velocity value (V, mm/s) 26% lower and a volume velocity value (Q, mm3/sec) 35% lower than did patients of 2^nd^ group. In 1^st^ group in comparison with control group reduction of V was 49% and reduction of Q was 57% while the difference between 2^nd^ group and controls was 32% in V and 39% in Q. The normal vascular response of gingiva vessels to cold test was found only in 63% controls. 100% of patients with 2>MetS factors developed the atypical vascular response to cold test while in 2^nd^ group it was determined in 69% of patients. The weakened vascular response was detected in 31% of patients in the 2^nd^ group and 27% of controls.

## Conclusions

Patients with more MetS components develop more pronounced periodontal destruction and gingiva microvascular dysfunction than patients with less number of MetS components. Patients with MetS have reduced blood flow velocity values (V>32%; Q>39%) in comparison with healthy controls.

## Outlook and expert recommendations

Patients with central obesity and even one MetS factor are recommended to have complex dental investigation and supportive periodontal treatment.
